# Book Reviews

**Published:** 1916-04

**Authors:** 


### BOOK REVIEWS

INTERNATIONAL CLINICS. Volume 1, Twenty-sixth Series. A Quarterly of Illustrated Clinical Lectures and Especially Prepared Original Articles on the General Subjects of Medicine and Surgery as well as the Specialties, by Leading Members of the Medical Profession throughout the World. Edited by H. R. M. Landis, M.D., of Philadelphia, with the Collaboration of many Leading Men. Cloth, 326 Pages and 64 Illustrations. Philadelphia and London: J. B. Lippincott Company.

The character of this publication has long been established. The plan of the editor and publishers to select and secure those articles that seemed to them to give the latest and the most careful thought in the advances of medicine has been sustained and we have before us a series of essays telling of the work and of the discoveries of the last few months. To describe each of them would go beyond a review's limits. They are all good and together, they extend over a wide field. A list of the contents will give the best idea to the intending purchaser of what the book means and does.

Under Treatment we have Chorea, including a New Treatment by E. E. and W. H. Mayer. Drug Therapy in Cardiovascular Disease, by Thomas E. Sattherwaite, and Treatment of Internal Tuberculosis by Means of Absorbed Light Energy, by Th. Brinck.

Medicine presents Pellagra, by Beverly R. Tucker; Early Diagnosis of Gastric Cancer by Julius Friedenwald, and Severe Secondary Syphilis, by E. P. Weber and Hans Schmidt.

In Neurology we have The Wounded Mind, by J. P. H. Murphy.

Public Health. The Relation of the Practicing Physician to Public Health Administration and the Registration of Births and Deaths, by John W. Trask.

Pathology. Some Regenerative Phenomena and their Possible Relation to Tumor Formation, by G. J. Saxon and Ellen P. Corson-White.

Gynecology. Prolapse of the Genital Organs in Women, by H. T. Byford. The Management of Inevitable Abortion, by C. L. Nichols.

Surgery. Spina Bifida and the Surgical Treatment of its More Serious Forms, by W. W. Babcock. Combined Efforts to Annulify Surgical Shock, by G. S. Foster. The Non-Operative Treatment of Long Bones, by J. B. Roberts. The Technic of Radium Treatment per Urethram in Prostatic Carcinoma, by Jean Cauhape. Infection of the Right Scrotum and Inguinal Region with Bacillus Aerogenes Capsulatus Simulating a Hernia, by J. F. Stewart.

A General Review of Medicine for the Year 1915, by F. A. Craig and John Speese.

---

A TREATISE ON MEDICAL PRACTICE based on the principles and therapeutic applications of The Physical Modes and Methods of Treatment. By Otto Juettner, A.M., Sc.M., Ph.D., M.D., Author of "Modern Physiotherapy, "Daniel Drake and His Followers;" Surgeon M. R. C., United States Army; Fellow of the Academy of Medicine of Cincinnati, of the American Medical Association, the American Therapeutic Society, the American Academy of Medicine, the American Medical Editors' Association, etc., etc. Octavo volume of 519 pages. New York: A. L. Chatterton Co., 1916. Cloth, $5.00.

At first sight of the title and with a cursory glance at some of the pages one has the feeling that the writer is developing a fad and projecting a special kind of practice to the exclusion of rational treatment and to the deprivation of the patient of what would be best suited to his needs.

A further study of the title and an analysis of the introductory pages, combined with an examination of the therapy the book suggests, changes the impression and discovers that the writer has taken due thought of his subject and is endeavoring to show his readers that they have been overlooking many valuable things in the care of the sick.

There is always danger that a man may make a fad of not having fads or of entertaining them, and he will place a possible stumbling block in his way. Thus it is that when an established practitioner is presented with a thesis upon special methods, he is likely to ignore it and do this to his loss. There are many things in this book to give that impression as has been said, but, at the same time, there are so many others that show the common sense of the writer and his wide experience with disease and all the modern methods for combatting it that the reader will soon give weight to all that is said and he will find himself considering whether or not these methods are not pretty valuable after all.

There is nothing especially new in any of them. There has been no attempt to set forth the difficult and unattainable means of non-medical treatment. The novel element is the dignified presentation in an authoritative tone of a long list of things that most of us have been looking at askance and generally avoiding.

The tone is authoritative and sometimes it is assertive to the extent of raising a question as to the author's right to speak, but we suppose he knew that and is confident of himself and his reputation to support strongly the ideas he presents for what they are worth.

The range of diseases to be treated either completely or in such measure as the medical attendant may elect by non-medical means is very extensive and comprehensive, and when reading the advice given, one should always keep in mind the clearly expressed intent of the author that these things are not exclusive in their application, but that they do have a highly important function.

The methods of applying electro-, hydro- and thermo-therapy are simple and require no great expenditure of money. The description is not pedantic and calculated to make one go to the author for special instruction. The chapter on diet and that on toxicity are excellent. There is an honest intent to stimulate every man to practise carefully and thoughfully the methods the author has proved to be good. This drives away any antagonistic early impressions and leaves the reader thinking and very likely planning.

The work is an excellent textbook for non-medical treatment. In form, while large, it is clear and concise in its diction and in the bookmaking the publishers have conferred the blessing of light weight and ease in handling.

It is commended to men who can thoughtfully analyze their methods and note their own empiricism in the ordinary practise; they are pretty certain to find valuable assistance in their work.

---

**CHAUFFEUR'S KNEE. By Gustav F. Boehme, Jr., B. S., M. D., New York, Neurologist, West Side German Dispensary; Assistant Physician, Neurological Institute, Etc.**

**For years we have sympathized with the patient who is on his feet for many hours daily, for he has been the victim of callouses and corns, of foot deformities and that bane of all clerks and store keepers, pes planus. A short time hence I called attention to "tango foot" brought on by the flights of the terpsichorean artist and devotee. At this time I desire briefly to call attention to a minor ailment occurring in those who drive automobiles to any extent, or who are not accustomed to this form of sport.**

A number of drivers of cars have in the past two years consulted me for a pain in the knee, made worse by ascending stairs or on moving the knee in the control of the pedals of their machine; this was their only complaint.

On examination flexion and extension of the knee (usually the right one) was limited and painful, and the patient was cognizant of a sense of grating in the front knee. On each side of the patella a fluctuating swelling, of more or less extent, could be noted, depending upon the length of time the condition had existed. The picture is one of a bursitis of the subtendinous bursa lying between the tendinous expansion of the quadriceps and the periosteum of the patella. The process differs from that of housemaid's knee in that the superficial bursa is uninvolved, and no swelling is felt in front of the quadriceps tendon in movement, but laterally to it the swellings can be felt to change in size, while the grating feeling is distinctly made out.

The mechanism of this process is as follows: The position of the knee in the ordinary car is a rather cramped one; flexion and extension of the knee occur in shifting the gas and other pedals by the foot. In those predisposed to this condition, this constant movement sets up a low grade inflammation in the subtendinous bursa.

What has been rather surprising is that in none of these cases has there been any sign of inflammation in the ankle joint or in the tendons crossing it. I am at a loss to explain this, yet I have never seen a tenosynovitis of the ankle tendons in a chauffeur. I have felt, however, that the fact that the ankle movement is not a cramped one, while that of the knee is undoubtedly limited by the position in the car seat, and thus adds in its semiflexion to the difficulty of moving the quadriceps tendon, occasions more pressure on the bursa than usual, and in this way predisposes to inflammation at this point.

Treatment, briefly summarized, consists of cessation from driving, rest for a short time, with local applications of aluminum subacetate or lead and opium solution. Passive movements with massage and baking should be begun early to prevent stiffness of the knee.

---

DIAGNOSTIC METHODS, A Guide for History Taking, Making of Routine Physical Examination and the Usual Laboratory Tests Necessary for Students in Clinical Pathology, Hospital Internes and Practicing Physicians.

By Herbert Thomas Brooks, A.B., M.D., Professor of Pathology, University of Tennessee, College of Memphis, Tennessee. Third Edition, Revised and Rewritten. 96 Pages, Illustrated. Cloth, $1.00. St. Louis, C. V. Mosby Co.

In this small and convenient book there are contained descriptions of the methods of laboratory examinations and tests that are clear and plain in themselves and so arranged as to avoid the least confusion in systematizing work. To the former editions there has been added a new chapter on the technique of staining and examining smears and the most important exudates. Besides this there has been eliminated everything that has either become obsolete or has proven to be questionable. There results just the kind of helpful and guiding book that the practitioners who desire to do much of their own laboratory work need and there is given to the worker, however experienced, a valuable epitome which he may have always at hand.

---

A HANDBOOK OF INFANT FEEDING. By Lawrence T. Royster, M.D., Attending Physician Bonney Home for Girls and Fondling Ward of the Norfolk Society for the Prevention of Cruelty to Children, Physician-in-Charge of the King's Daughters' Visiting Nurse Clinic for Sick Babies. 144 Pages, Illustrated. Cloth, $1.25. St. Louis: C. V. Mosby Co.

The author states that the purpose of the book is to furnish the essentials and only the essentials of infant feeding in a compact and succinct form. The number of books that have come out recently upon this subject show that the Profession is demanding information the general practitioner can readily use, and one examines the book to see whether the purpose has been filled and the demand met.

The ground has been well covered and the reader can be confident that he will find valuable and carefully considered information in each of the chapters. It is a book for everyday, household practise.

